# Risk factors for late reconnections after circumferential pulmonary vein isolation guided by lesion size index – Data from repeat invasive electrophysiology procedure

**DOI:** 10.3389/fcvm.2022.986207

**Published:** 2023-01-26

**Authors:** Nebojša M. Mujović, Milan M. Marinković, Nebojša Marković, Aleksandar Kocijančić, Vladan Kovačević, Vera Vučićević, Nataša M. Mujović, Tatjana S. Potpara

**Affiliations:** ^1^Cardiology Clinic, University Clinical Center of Serbia, Belgrade, Serbia; ^2^Faculty of Medicine, University of Belgrade, Belgrade, Serbia; ^3^Mount Sinai Heart, Icahn School of Medicine at Mount Sinai, New York, NY, United States; ^4^Center for Anesthesiology, University Clinical Center of Serbia, Belgrade, Serbia; ^5^Center for Physical Medicine and Rehabilitation, University Clinical Center of Serbia, Belgrade, Serbia

**Keywords:** atrial fibrillation, catheter-ablation, pulmonary vein isolation, pulmonary vein reconnection, lesion size index, WACA, durability

## Abstract

**Background:**

Late reconnections (LR) of pulmonary veins (PVs) after wide antral circumferential ablation (WACA) using point-to-point radiofrequency (RF) ablation are common. Lesion size index (LSI) is a novel marker of lesion quality proposed by Ensite Precision mapping system, expected to improve PV isolation durability. This study aimed to assess the durability of LSI-guided PVI and the risk factors for LR of PVs.

**Methods:**

The prospective study included 33 patients with paroxysmal atrial fibrillation (PAF) who underwent (1) the index LSI-guided WACA procedure (with target LSI of 5.5-6.0 for anterior and 5.0-5.5 for posterior WACA segments) and (2) the 3-month protocol-mandated re-mapping procedure in all patients, irrespective of AF recurrence after the index procedure. Ablation parameters reported by Ensite mapping system were collected retrospectively. The inter-lesion distance (ILD) between all adjacent WACA lesions was calculated off-line. Association between index ablation parameters and the LRs of PVs at 3 months was analyzed.

**Results:**

The median patient age was 61 (IQR: 53–64) years and 55% of them were males. At index procedure, the first-pass WACA isolation rate was higher for the left PVs than the right PVs (64 vs. 33%, *p* = 0.014). In addition, a low acute reconnection rates were observed, as follows: in 12.1% of patients, in 6.1% of WACA circles, in 3.8% of WACA segments and in 4.5% of PVs. However, the 3-month remapping study revealed LR of PV in 63.6% of patients, 37.9% of WACA circles, 20.5% of WACA segments and 26.5% of PVs. The LRs were identified mostly along the left anterior WACA segment. Independent risk factors for the LR of WACA were left-sided WACA location (OR 3.216 [95%CI: 1.065–9.716], p = 0.038) and longer ILD (OR 1.256 [95%CI: 1.035–1.523] for each 1-mm increase, p = 0.021). The ILD of > 8.0 mm showed a predictive value for the LR of WACA, with the sensitivity of 84% and specificity of 46%. A single case of cardiac tamponade occurred following the re-mapping invasive procedure. No other complications were encountered.

**Conclusion:**

Although the LSI-guided PVI ensures a consistent PVI during the index procedure, LRs of PVs are still common. Besides the LSI, the PVI durability requires an optimal ILD between adjacent lesions, especially along the anterior lateral ridge.

## Introduction

Pulmonary vein isolation (PVI) using wide antral circumferential ablation (WACA) guided by 3D mapping system is a cornerstone of catheter-ablation (CA) for paroxysmal atrial fibrillation (PAF) (1). However, achieving durable PVI is challenging, requiring multiple procedures in 20-40% of patients (1). A long-term quality of WACA lesion using point-to-point ablation is related to many factors, including catheter tip-to-tissue contact force (CF), power output, catheter stability, time of radiofrequency (RF) delivery, distance between neighboring lesions and atrial wall thickness (2). If the parameters that ensure transmurality and continuity of WACA lesion(s) were determined, it could improve the PVI durability after single CA.

The CF measuring catheters facilitate creation of more effective lesions (3, 4). Although the force-time integral (FTI)-guided ablation increases PVI durability compared with routine CF-guided ablation, 40% of the patients still exhibited late reconnections (LR) of pulmonary veins (PVs) (4). The lesion size index (LSI) is a novel marker of lesion quality that combines FTI and applied power, and has been shown to strongly correlate with RF lesion width and depth *in vitro* (5). Recently, the AutoMark module of the Ensite mapping system (Abbott, USA) for automated lesion tagging has been proposed to generate a tag only if prespecified requirements during lesion formation are met, thus providing an opportunity for better standardization and reproducibility of the PVI procedure (6). Although these technological advancements are expected to reduce LR of PVs, the PVI durability after LSI-guided WACA using the AutoMark software for automated lesion tagging is not reported yet.

The study objectives were: (1) to determine the durability of the index LSI-guided PVI performed with assistance of the AutoMark software, and assessed by the 3-month protocol-mandated invasive remapping procedure in patients with PAF, and (2) to evaluate the risk factors for the LR of PVs.

## Materials and methods

### Patient selection

This prospective, single-center study was conducted in Clinical Center of Serbia. Of 148 consecutive CA procedures for AF, performed between November 2017 and January 2019, 115 procedures were excluded from further analysis due to non-paroxysmal AF (*n* = 35), substrate ablation beyond PVI (*n* = 17), re-do CA (*n* = 25), PVI not guided by LSI (*n* = 37) or patient refusal of the follow-up invasive procedure (*n* = 1).

Therefore, the study group (*n* = 33) consisted of patients with PAF, who underwent the two-step ablation strategy, consisting of the index LSI-guided PVI as a stand-alone CA treatment and the 3-month repeat invasive procedure. All patients provided written informed consent and the study protocol was approved by the hospital ethics committee.

### The index PVI procedure

All patients received optimal preprocedural anticoagulation for 4-6 weeks and all antiarrhythmic drugs (AADs) were discontinued for at least 3-5 half-lives before CA (1). The invasive procedure was performed under a deep sedation with propofol, midazolam, fentanyl and/or ketamine. The procedure strategy was previously presented in details elsewhere (7). After selective PV angiography, the left atrial (LA) geometry was collected by circular duo-decapolar catheter (20 mm diameter, AF Focus II, Abbott, USA) and electro-anatomical mapping system (Ensite Precision, Abbott, USA); afterward, the 3D map was integrated with the segmented computed tomography (CT) image of the LA. The esophagus was segmented from the CT scan to visualize its relation to the posterior LA and to optimize selection of the ablation trajectory.

#### The WACA circle ablation

**FIGURE 1:**
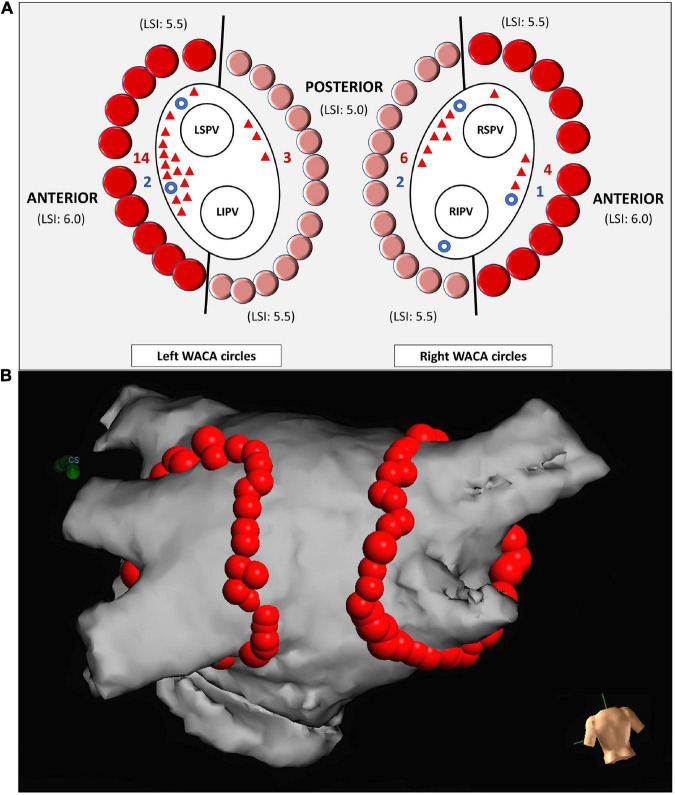
Schema representing ipsilateral PV isolation by left and right WACA circles and the sites of reconnections **(A)**. Each WACA circle was anatomically divided into the anterior segment (dark red dots) and the posterior segment (light red dots). The point-to-point LSI-guided ablation of the anterior segment was performed with target LSI from 5.5 (superior region of WACA) to 6.0 (anterior region of WACA). Ablation of the posterior segment was performed with target LSI from 5.0 (posterior region of WACA) to 5.5 (inferior region of WACA). The sites of the acute and the late WACA segment reconnections are illustrated by blue circles and red triangles, respectively. The posterior view of the left atrium **(B)**. The lesion tags were annotated automatedly by the AutoMark software of the Ensite Precision mapping system, as the 3D red balls. The tag size was 5 mm in diameter for lesions reaching LSI 5.0-5.5, and 6 mm for those with LSI > 5.5. LSI, lesion size index; LSPV, left superior pulmonary vein; LIPV, left inferior pulmonary vein; RSPV, right superior pulmonary vein; RIPV, right inferior pulmonary vein; WACA, wide antral circumferential ablation.

Ipsilateral PVs were isolated in pairs by WACA circle lesions, consisting of adjacent point-by-point RF lesions deployed approximately 1-2 cm away from PV ostia, Figure 1. Initially, each WACA circle was divided into the 2 segments: the “anterior” segment, including superior and anterior WACA regions, and the “posterior” segment, consisting of inferior and posterior WACA regions, Figure 1A. During WACA ablation, residual PV activity was monitored using circular catheter. Ablation started at superior WACA region and continued along the predetermined WACA line, encircling both ipsilateral PVs. The ablation end-point was entrance and exit PV-LA block, confirmed by usual pacing maneuvers (7). If both ipsilateral PVs were not isolated during the first-pass WACA, further ablation was guided by PV activity on the circular catheter. The isolation status of ipsilateral PVs was re-evaluated using circular catheter 30 minutes after their initial isolation. All acute PV(s) reconnections were re-mapped and closed by additional regional ablation. The patients with history of typical atrial flutter (AFL) underwent cavo-tricuspid isthmus ablation (1).

#### The lSI-guided ablation

Ablation was performed using CF ablation catheter (TactiCath Quartz, Abbott, USA), which was navigated by steerable long sheath (8.5 Fr, Agilis NT medium curl, Abbott, USA). The ablation set-up was 30-35W and 25-30W, on the anterior and the posterior LA wall, respectively, with irrigation rate of 17 ml/min and with the CF target between 5-30 gr. The LSI formula integrates the data on CF, applied power and duration of RF delivery (6). Regional ablation was guided by prespecified LSI target of 5.5-6.0 and 5.0-5.5 along the “anterior” and the “posterior” WACA segments, respectively (8, 9), as illustrated in Figure 1A. If LSI target was not achieved (due to low CF, catheter instability or patient’s movements), ablation at the same site was repeated; FTI data during ablation were monitored but were not targeted. RF delivery was terminated immediately in case of impedance drop > 18 (Ω) or any impedance rise (10). The strategy to prevent esophageal damage included a modification of WACA trajectory at posterior LA wall according to esophagus location determined by CT (11), reduction of RF power to 20-25W, shortening of RF delivery time to 20 s, limitation of LSI to 5.0, decreasing CF to < 20 g (12) and/or abrupt termination of RF delivery if pain occurs (1).

The lesion tags were annotated automatedly by the AutoMark module of Ensite Precision mapping system, and only if pre-defined requirements on LSI were met. The tag size was 5 mm in diameter for lesions reaching LSI between 5.0 and 5.5, and 6 mm for those with LSI > 5.5, see Figure 1B. Following AutoMark settings were used: the lesion spacing = 6 mm, away time = 3 s and minimum lesion time = 8 s. After the AutoMark generated a tag at the site of “successful” ablation, application of RF current was terminated and catheter tip was moved approximately 4-5 mm along intended WACA circle, to start a new ablation and to create a new adjacent lesion.

### The repeat electrophysiology procedure

The invasive re-mapping procedure was performed 3 months after the index CA. Preprocedural preparing and ablation strategy were the same as in the index CA. All 4 PVs were re-evaluated using circular catheter for the entrance and the exit block, and the number and the sites of possible WACA circle/segment LRs were recorded. Finally, all LRs were closed by touch-up ablation, regardless of the arrhythmia recurrence between the procedures.

### Analysis of the ablation lesion parameters

A retrospective analysis of relationship between the index CA lesion data and the LR of WACA included whole study cohort (*n* = 33) and all tags along the WACA lesions (*n* = 2179). For each patient, the lesion tag sequence in the AutoMark data report was re-coded according to lesions’ visual sequence along the WACA circle(s) on the 3D map from index CA, to ensure the analysis of neighboring lesion tags along the ablation line. Lesion tags of additional RF ablations, applied to close acute WACA reconnections during the index procedure, were included in the analysis of LRs. For each lesion tag, following data were collected from the AutoMark report: mean RF power (W), mean CF (g), mean impedance drop (Ω), FTI (gs) and LSI.

#### Calculation of inter-lesion distance

**FIGURE 2:**
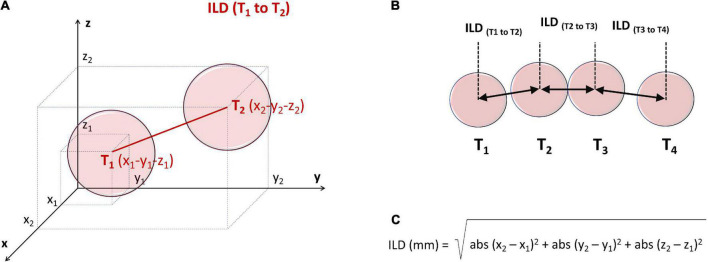
Principles of off-line measurement of ILD between the centers of two contiguous ablation lesion tags, T1 and T2 **(A,B)**, using the 3D Pythagorean Theorem equation **(C)**. For each lesion tag the AutoMark module reported x-y-z coordinates (on 3 orthogonal axes) from the reference system point. ILD, inter-lesion distance.

For each lesion tag the AutoMark module reported x-y-z coordinates (on 3 orthogonal axes) from the reference system point. Inter-lesion distance (ILD) between centers of two neighboring lesions was calculated off-line, using the Pythagorean Theorem formula for the 3D coordinate system, as presented in Figures 2A–C.

Finally, the “weakest” values of the ablation parameters were determined for each WACA circle and for each WACA segment, as those tags exhibiting a minimum RF power, minimum CF, minimum impedance drop, minimum FTI, minimum LSI or maximum ILD.

### Follow-up after CA

The AAD and oral anticoagulant drug that were used before the CA were administered during the 3-month blanking period post-CA in all patients. Afterward, AADs were discontinued in all patients, while anticoagulation was continued in those with a CHA_2_DS_2–_VASc score of > 1 (1).

The follow-up rhythm monitoring consisted of serial 24h Holter-recordings at discharge, 1, 3, 6 and 12 months post-CA, and every 6 months thereafter. Any (symptomatic or asymptomatic) documented atrial arrhythmia after CA, such as AF, atrial tachycardia (AT) or AFL, sustaining > 30 sec and re-occurring within the first 3 months post-CA or after 3 months post-CA, was considered as early or late recurrence of atrial arrhythmia (ERAA and LRAA), respectively (1, 7).

### Statistics

Continuous variables are presented as mean ± Standard Deviation or median with 25th and 75th percentiles, while categorical variables are given as percentages. Continuous variables are compared using independent t-test or Mann-Whitney test, as appropriate, and differences between proportions were evaluated by Chi-squared test or Fisher’s test. A cut-off value of continuous variables was determined using Receiver Operating Characteristic (ROC) analysis. Association between the index CA parameters and late WACA reconnection was initially assessed using univariate logistic regression analysis and then all variables with *p*-value of < 0.10 were included in the multivariate model, reporting Odds Ratio (OR) and Confidence Interval (CI). A two-sided *P*-value of < 0.05 was considered as significant. Statistics was conducted using the SPSS software (version 20.0, IBM, USA).

## Results

The study group included 33 patients (median age 61, IQR: 53 – 64 years); 18 patients (55%) were males. Baseline clinical characteristics of the patients are presented in Table 1.

**TABLE 1:** Baseline clinical characteristics of the study patients.

	All patients (*n* = 33)	Patients with late WACA reconnection (*n* = 21)	Patients without late WACA reconnection (*n* = 12)	*P*-value
Age (years)	61 (53–64)	62 (52–65)	60 (52–64)	0.567
Male sex	18 (55%)	15 (71%)	3 (25%)	0.014
BMI (kg/m^2^)	26 ± 4	27 ± 4	26 ± 5	0.942
Pre-CA history of AF (years)	4.5 ± 3.2	4.9 ± 3.6	3.9 ± 2.3	0.391
LA diameter (mm)	40 ± 4	40 ± 4	39 ± 3	0.475
Left ventricular EF (%)	60 (59–62)	60 (60–62)	60 (58–62)	0.671
Hypertension	25 (76%)	15 (71%)	10 (83%)	0.678
Ischemic heart disease	1 (3%)	1 (5%)	0	>0.999
Diabetes mellitus	3 (9%)	2 (10%)	1 (8%)	>0.999
Congestive heart failure	1 (3%)	0	1 (8%)	0.364
CHA_2_DS_2_VASc score	2 (1–2)	1 (0 – 2)	2 (1 – 2)	0.200
GFR (ml/min)	94 ± 27	97 ± 29	89 ± 22	0.482
Number of failed Class IC/III AADs	2.3 ± 0.9	2.1 ± 0.9	2.7 ± 0.9	0.119
Amiodarone before CA	18 (55%)	11 (52%)	7 (58%)	0.741
Beta-blocker before CA	26 (79%)	16 (76%)	10 (83%)	>0.999
Typical flutter ablation	10 (30%)	6 (29%)	4 (33%)	>0.999
Fluoroscopy time (min)	15.1 ± 5.1	15.8 ± 4.9	13.8 ± 5.5	0.303
ERAA after index CA	6 (18%)	5 (24%)	1 (8%)	0.379

Data are presented as mean ± SD, median (with quartiles) or percentages. WACA, wide antral circumferential ablation; BMI, body mass index; CA, catheter-ablation; AF, atrial fibrillation; LA, left atrium; EF, ejection fraction; GFR, glomerular filtration rate; AAD, antiarrhythmic drug; ERAA, early recurrence of atrial arrhythmia.

### The index CA procedure

A common left PV trunk was identified in 6 patients (18%), while right-sided PVs’ ostia were clearly separated in all patients. The ablation was started during sinus rhythm (*n* = 23) or AF/AFL/AT (*n* = 10).

The region-prespecified LSI was achieved in 95% (IQR: 90 – 98) of all lesions along the WACA. The first-pass ipsilateral PVs isolation rate was two-fold higher among the left-sided than among the right-sided PVs pairs (64 vs. 33%, *p* = 0.014). All PVs were isolated.

There were no significant differences between the left- and right-sided WACA circles with respect to majority of ablation parameters presented in Table 2 (all *p* > 0.05), except that the ablation time was significantly higher for the left-sided, while the CF and LSI were significantly higher for the right-sided WACA circles.

**TABLE 2:** Ablation data from the index procedure.

	WACA circles (*n* = 66)			WACA segments (*n* = 132)		
	Left-sided WACA (*n* = 33)	Right -sided WACA (*n* = 33)	*P*-value	Anterior segments (*n* = 66)	Posterior segments (*n* = 66)	*P*-value
First-pass isolation of ipsilateral PVs	21 (64%)	11 (33%)	0.014	-	-	-
Number of RF tags	32 ± 7	34 ± 6	0.350	33 (28-38)	27 (23–31)	<0.001
WACA length (mm)	138 ± 25	145 ± 25	0.231	75 ± 17	66 ± 16	0.002
RF power (W)	31 ± 1	31 ± 2	0.283	32 (31–33)	30 (28–32)	<0.001
RFtime (sec)	32 (27–36)	25 (22–28)	<0.001	33 (28–39)	22 (19–25)	<0.001
CF(g)	15 (12–18)	18 (16–19)	0.003	16 ± 4	17 ± 5	0.042
Impedance drop (Ω)	14 ± 2	13 ± 2	0.314	14 ± 2	13 ± 2	<0.001
FTI (gs)	418 ± 80	409 ± 79	0.660	463 (406–525)	358 (307–401)	<0.001
LSI	5.5 (5.4–5.6)	5.6 (5.5–5.7)	0.005	5.8 (5.6–5.9)	5.3 (5.2–5.4)	<0.001
ILD (mm)	4.3 (3.9-4.6)	4.2 (3.8–4.9)	0.903	4.3 (3.9–4.8)	4.1 (3.6–4.7)	0.203

Data are presented as mean ± SD, median (with quartiles) or percentages. WACA, wide antral circumferential ablation; PV, pulmonary vein; RF, radiofrequency; CF, contact force; FTI, force-time integral; LSI, lesion size index; ILD, inter-lesion distance.

A more aggressive ablation strategy along the anterior (vs. the posterior) LA wall is reflected by a significantly higher number of RF lesions, higher RF power, a longer ablation time, a more pronounced impedance drop, and higher FTI and LSI (per lesion) on the anterior compared with the posterior WACA segments (all *p* < 0.001, see Table 2).

The 30-minute acute PV reconnections were observed in 4/33 patients (12.1%), 4/66 WACA circles (6.1%), 5/132 WACA segments (3.8%) and 6/132 PVs (4.5%). The anatomical distribution of the WACA segments with acute reconnection was as follows: 2 left-sided anterior segments, 2 right-sided posterior segments and 1 right-sided anterior segment. The distribution of PVs exhibiting acute reconnection was as follows: left superior PVs 2, right superior PVs 2, left inferior PV 1 and right inferior PV 1. All acute reconnections were closed by touch-up ablation resulting in the mean total RF delivery time for PVI of 31.7 ± 6.5 min. Subsequent ablation of typical AFL (n = 10) or AVNRT (n = 1) was successful in all patients and total fluoroscopy procedure time was 15.1 ± 5.1 min. There were no major periprocedural complications, including fatal outcome, cardiac tamponade, stroke, groin hematoma and esophageal damage.

The ERAA was detected in 6 patients (18.2%) in the form of PAF in all of them, after 16 (IQR: 2-34) days following the CA.

### The repeat electrophysiology procedure

The median time from index to repeat invasive procedure was 112 (IQR: 105 – 132) days.

The LR (of PVs) was identified in 21/33 patients (63.6%), 25/66 WACA circles (37.9%), 27/132 WACA segments (20.5%) and 35/132 PVs (26.5%). The median number of reconnected PVs per patient was 1.0 (IQR: 0.0 – 2.0) and the distribution of PVs exhibiting the LR was as follows: left superior PVs 14 (10.6%), left inferior PVs 10 (8.6%), right superior PVs 6 (4.5%) and right inferior PVs 5 (3.8%). Majority of reconnected WACA segments (24/27 [88.9%]) exhibited only 1 conduction gap, except 3 segments (left anterior, left posterior and right posterior segment) showing 2 gaps each.

**FIGURE 3:**
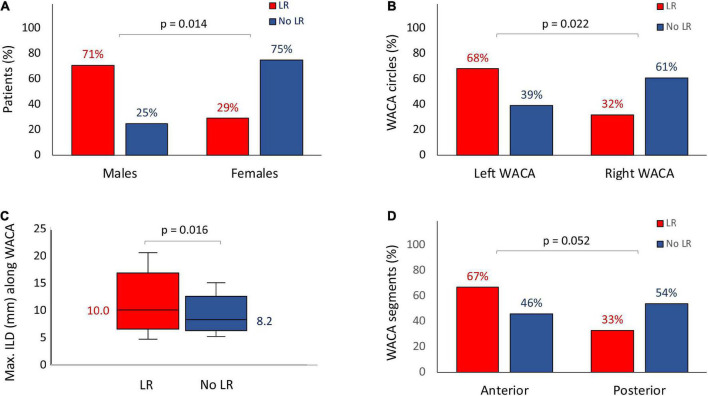
Risk factors for the late WACA circle/segment reconnection: the male gender **(A)**, the left-sided location of WACA circle **(B)**, the maximum ILD along WACA circle **(C)** and the anterior segment of WACA **(D)**. All abbreviations as in previous figures.

The LR of WACA circles were significantly more common among males than among females (15/21 [71%] vs. 6/21 [29%], p = 0.014) as well as among the left-sided compared with the right-sided WACA lesions (17/25 [68%] vs. 8/25 [32%], *p* = 0.022), see Table 1 and Figure 3. In addition, the LR tended to be more common along the anterior than posterior WACA segments (18/27 [67%] vs. 9/27 [33%], *p* = 0.052).

All reconnected PVs were successfully re-isolated by RF ablation, irrespective of previous clinical arrhythmia relapse. The total RF ablation time and fluoroscopy time were 5.0 (IQR: 2.7 – 7.1) min and 10.9 (IQR: 7.6 – 13.1) min, respectively. A single case of cardiac tamponade occurred following left PVs re-isolation and the patient has fully recovered after percutaneous pericardial drainage. No other major complications were encountered (see Table 3).

**TABLE 3:** Risk factors for the WACA segment reconnection.

	WACA segments (*n* = 132)					
	Univariate analysis				Multivariate analysis	
	With LR (*n* = 27)	Without LR (*n* = 105)	OR (95% CI)	*P*-value	OR (95% CI)	*P*-value
Left-sided WACA segments	17 (63%)	49 (47%)	1.943 (0.814–4.638)	0.135	-	-
Anterior WACA segments	18 (67%)	48 (46%)	2.375 (0.978–5.769)	0.056	-	-
Number of RF tags (n)	30 ± 9	30 ± 7	1.010 (0.955–1.068)	0.731	-	-
WACA segment length (mm)	76 (63–85)	67 (55–81)	1.024 (0.999–1.050)	0.060	-	-
Min. RF power (W)	28 (27–28)	28 (27–28)	0.988 (0.838–1.164)	0.884	-	-
Min. RF time (sec)	11 (9–15)	11 (9–14)	0.980 (0.893–1.076)	0.673	-	-
Min. CF (g)	7 (7–10)	8 (6–9)	0.995 (0.857–1.155)	0.948	-	-
Min. Impedance drop (Ω)	8 (6–9)	7 (5–8)	1.181 (0.960–1.453)	0.115	-	-
Min. FTI (gs)	188 (115–269)	185 (125–245)	1.000 (0.996–1.004)	0.965	-	-
Min. LSI	4.6 (3.8–5.1)	4.7 (3.8–5.0)	0.923 (0.557–1.529)	0.755	-	-
Max. ILD (mm)	8.5 (7.3–11.0)	7.5 (6.5–9.0)	1.147 (0.996–1.320)	0.057	-	-
Max. ILD > 8.0 mm	18 (67%)	43 (41%)	2.884 (1.185–7.020)	0.020	2.884 (1.185–7.020)	0.020

Data are presented as mean ± SD, median (with quartiles) or percentages. All abbreviations as in previous tables.

### Risk factors for the LR

#### The LR of WACA circle

**FIGURE 4:**
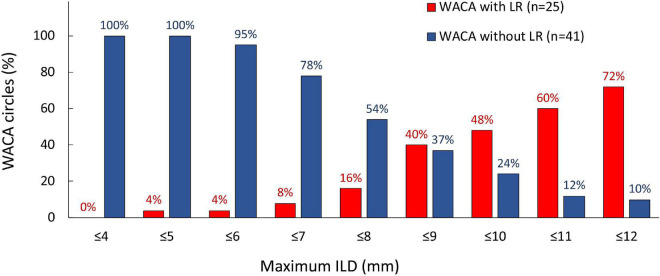
Distribution of the maximum ILD according to the 3-month isolation status of WACA circles. On the cut-off analysis, a maximum ILD of > 8.0 mm showed significant predictive value for the late WACA reconnection, with sensitivity 84% and specificity 46%. In > 95% of isolated WACA circles the maximum ILD was < 6 mm, while > 40% of WACA circles with late reconnection had the maximum ILD > 8 mm. All abbreviations as in previous figures.

On univariate analysis, the LR of WACA circle was significantly associated with left-sided location of WACA and maximum ILD (mm) along WACA circle, Table 4. The association was also significant on multivariate analysis: OR 3.216 [95%CI: 1.065 – 9.716], p = 0.038 for the left-sided WACA, and OR 1.256 [95%CI: 1.035 – 1.523] for each 1 mm increase, p = 0.021) for maximum ILD along the WACA. The maximum ILD showed moderate predictive ability for the LR of WACA circle (area under curve - AUC 0.686 [95%CI: 0.552 – 0.821], p = 0.012). On the cut-off analysis, a maximum ILD of > 8.0 mm had a sensitivity of 84% and specificity of 46% in predicting the LR of WACA circle. Figure 4 illustrates the relationship between the maximum ILD and the 3-month isolation status of WACA circles. Thus, in > 95% of isolated WACA circles the maximum ILD was ≤ 6 mm, while > 40% of WACA circles with LR had the maximum ILD of > 8 mm.

**TABLE 4:** Risk factors for the late WACA circle reconnection.

	WACA circles (*n* = 66)					
	Univariate analysis				Multivariate analysis	
	With LR (*n* = 25)	Without LR (*n* = 41)	OR (95% CI)	*P*-value	OR (95% CI)	*P*-value
First-pass WACA isolation	9 (36%)	23 (56%)	0.440 (0.158–1.225)	0.116	-	-
Left-sided WACA	17 (68%)	16 (39%)	3.320 (1.163–9.477)	0.025	3.216 (1.065–9.716)	0.038
Number of RF tags (n)	32 ± 8	33 ± 6	0.982 (0.913–1.057)	0.635	-	-
Percent of RF tags with achieved LSI target	93 (90–98)	96 (90–98)	0.990 (0.938–1.046)	0.729	-	-
WACA length (mm)	145 ± 24	139 ± 26	1.010 (0.990–1.031)	0.324	-	-
Min. RF power (W)	27 (25–27)	27 (26–28)	0.761 (0.546–1.060)	0.106	-	-
Min. RF time (sec)	9 (9–10)	9 (8–11)	0.919 (0.715–1.182)	0.510	-	-
Min. CF (g)	7 (69)	6 (5–8)	1.163 (0.930–1.453)	0.186	-	-
Min. Impedance drop (Ω)	6 (5–8)	5 (5–8)	1.201 (0.920–1.567)	0.179	-	-
Min. FTI (gs)	142 (82–193)	130 (80–197)	1.001 (0.994–1.007)	0.852	-	-
Min. LSI	3.9 (3.2–4.4)	4.1 (3.5–4.7)	0.684 (0.373–1.256)	0.221	-	-
Max. ILD (mm)	10.0 (8.3–13.0)	8.2 (7.3–10.0)	1.269 (1.046–1.539)	0.016	1.256 (1.035–1.523)	0.021
Max. ILD > 8.0 mm	21 (84%)	22 (54%)	4.534 (1.321–15.557)	0.016	-	-

Data are presented as mean ± SD, median (with quartiles) or percentages. LR, late reconnection; OR, Odds Ratio; CI, Confidence Interval. Other abbreviations as in previous tables.

#### The LR of WACA segments

Although univariate analysis showed potential association between the LR of WACA segments and several factors, such as the anterior location of segment, segment length, maximum ILD (mm) and presence of maximum ILD > 8.0 mm along the segment (Table 4), a multivariate analysis identified the maximum ILD of > 8.0 mm within the segment (OR 2.884 [1.185 – 7.020], *p* = 0.020) as single independent risk factor for the LR of WACA segment.

### Clinical follow-up after ablation treatment

During the follow-up of 17 ± 5 months after the repeat procedure, class I/III AAD therapy was re-introduced in 6 patients (flecainide in 4 and amiodarone in 2) due to detected ERAA (*n* = 3), frequent ventricular premature beats (*n* = 2) or LRAA (*n* = 1), while 26 patients received a beta-blocker. All 12 patients with durable PVI after the first ablation procedure were free from AF during the follow-up. After the invasive EP study was repeated and all of the PV reconnections were closed by second ablation (*n* = 21 patients), the freedom from AF was 32/33 (97%).

## Discussion

This prospective study of 33 patients with PAF who underwent a structured invasive treatment strategy consisting of the index LSI-guided WACA and 3-month protocol-mandated re-mapping procedure demonstrated the LR (of PVs) in 64% of patients and in 38% of WACA circles. The LR of WACA was independently associated with the left-sided location of WACA lesion and maximum ILD between neighboring lesions along the WACA circle. The maximum ILD of > 8 mm showed a significant predictive value for the LR.

### Markers of lesion quality and durability of PVI

Achieving the continuous and transmural WACA lesion(s) for PVI in a single CA procedure using point-by-point RF ablation is challenging and depends on several factors, including CF, power output, catheter stability during RF delivery, time of RF delivery, contiguity of adjacent lesions and wall thickness (2).

At constant power and duration of RF delivery, CF is a critical determinant of lesion size (13). Although the CF-measuring catheters (vs. non-CF-sensing catheters) improved the effectiveness of PVI procedure, a significant proportion (62%) of patients still exhibited the LR of PVs as a substrate for AF relapse (3, 4). In order to further improve PVI procedure several novel markers of RF lesion quality, such as FTI, LSI and ablation index (AI) were recently proposed (2–4, 8, 9, 14). The FTI integrates the total CF applied over the time of RF delivery (2). *In vitro* study demonstrated a good linear correlation between FTI and RF lesion size (5). In addition, the PVI using constant FTI target of > 400 gs almost doubled the proportion of patients with permanently isolated PVs, compared with routine non-CF-sensing PVI procedure (65% vs. 35%), but with an excess in cardiac perforation rate (8%) (4).

The indices using weighted formulas (i.e., LSI and AI) which incorporate CF, ablation time and power, *in vitro* correlate better with lesion dimension than FTI (5, 15). The minimum AI along WACA circle was independently predictive of durable PVI (16). Recently, the true durability of the AI-guided PVI, targeting AI of > 400 at the posterior and AI of > 500 at the anterior LA wall, was tested prospectively in patients with persistent AF (17). At the 2-month protocol-mandated repeat electrophysiology study, all PVs were isolated in 78% of patients (17). In addition, small clinical studies demonstrated that PVI guided by AI or LSI provides a low rate of acute/dormant PV reconnections (2-14%) (8, 18) and an excellent mid-term single-procedure clinical success (> 90%) among PAF patients (9, 18).

The PVI is very demanding procedure, and its success, safety and duration significantly depend on operator experience (19). In addition, manual annotation of lesion tags during PVI is prone to subjective assessment of catheter stability and position during RF delivery. In order to make PVI procedure more reproducible, two automated lesion tagging softwares (e.g., the Visitag by CARTO - Biosense Webster, USA and the AutoMark by Ensite Precision – Abbott, USA) based on catheter stability were developed (6). These modules annotate a new tag only if predefined criteria during ongoing ablation session are met, ensuring a more standardized procedure (6).

### The lSI-guided PVI

To the best of our knowledge, we were the first to analyze true durability of LSI-guided PVI, performed with assistance of the AutoMark software for automated lesion tag annotation (8, 9). According to previous clinical study and non-uniformity of LA wall thickness, our ablation strategy targeted LSI of 5.5-6.0 and 5.0-5.5 at thicker anterior/superior (2.8 ± 1.1 mm) and thinner posterior/inferior WACA segment (1.7 ± 0.8 mm), respectively (8, 9, 20).

Although LSI-guided PVI in our study confirmed a very low acute WACA and PV reconnection rate (app. 5% each), the rates of subsequent LR (of PVs) among patients, WACA circles and PVs were considerably higher - 64%, 38% and 27%, respectively. However, majority of reconnected WACA segments exhibited only single conduction gap, typically at the left lateral ridge and the right posterior WACA segment, reflecting a quite consistent previous ablation for the most remaining part of WACA circles. The independent risk factors for the LR of WACA were the left-sided WACA location and a longer maximum ILD (mm) along WACA, increasing the LR probability by 3.2-fold and 1.25-fold, respectively. In addition, reconnections were more common among males and along anterior WACA segments. These findings are not surprising, because the left lateral ridge is the thickest part of muscular LA wall, reaching > 4 mm in thickness (20). Moreover, CF during PVI is significantly lower at all positions in the left PVs (vs. right PVs), especially at the left lateral ridge (3). When CF is weak, a longer RF delivery time is required to achieve the targeted LSI (2). However, keeping the catheter in a stable position at the lateral ridge during extended RF delivery can be very challenging, potentially affecting the lesion quality (1).

The creation of continuous ablation line by point-to-point RF ablation requires not only an optimal quality of each lesion along the line, but also an optimal distance between adjacent lesions (2, 16). Ablation lesions have an ellipsoid shape with a diameter up to 70% greater than their depth, and the lesion diameter is smaller at superficial (endocardial and epicardial) surfaces than deeper within the myocardium. Therefore, a substantial overlapping of neighboring lesions is a prerequisite for the line continuity and this could be even more important in the thicker LA wall regions (2).

The distance between delivered lesions is a critical determinant of histological continuity of ablation line *in vitro* and no gaps should remain when the visual lesion separation is < 1.5 mm above the mean LSI of adjacent lesions (21). Clinical study recognized the maximum ILD (between centers of two adjacent lesions) as an independent risk factor for the LR of PVs/WACA, with significantly better PVI/WACA durability among patients with maximum ILD < 6 mm than among those with wider inter-lesion gaps (16). The “CLOSE” protocol for PVI (CARTO, Biosense Webster), that combines the AI regional targets - guided PVI and the maximum ILD of < 6 mm along WACA circle, promoted a very high rate of first-pass ipsilateral PVI (98%), low acute PV reconnection rates (3%), and excellent 1-year single-procedure rhythm outcome (94%) (22).

The current version of the Ensite Precision does not provide real time ILD data during ablation. In our study, the ILD were retrospectively calculated, using a simple mathematical formula. The maximum ILD of > 8 mm arose as a high-sensitive but low-specific marker of the LR, because there is a lot of overlap in maximum ILD between the patients who exhibited and those not exhibiting the reconnection. This finding may be explained by several factors. First, pre-existing fibrosis of unablated areas along intended WACA line could facilitate the isolation of WACA segments, in spite of substantial inter-lesion gap(s) (23, 24). In addition, the AutoMark software displays the 3D ball tags with a preselected diameter (e.g., 4 mm, 5 mm, or 6 mm) which usually does not exactly match the LSI achieved, thus potentially affecting visual assessment of the ablation line continuity. Furthermore, visual gaps along WACA segments superimposed over the esophagus were skipped because of safety issues. Finally, the patients with “wider” gaps along the WACA (e.g., ILD > 6 mm) or “non-optimal” WACA circle(s) were excluded from the analysis in previous studies (16, 17), but not in our study. We believe that future update(s) of AutoMark software should provide a real time ILD feedback to operator during catheter re-positioning along the ablation line and the ability to select lesion tag size in accordance with the LSI achieved. These improvements could further increase the LSI-guided PVI durability. In our study, visual consistency of WACA lesions ensured a low acute PV reconnection rate at index CA. However, non-availability of real-time ILD data during ablation resulted in suboptimal ILD along WACA and substantially higher LR rate, probably due to resolution of edema between adjacent lesions in the days and weeks after the procedure (25).

### Limitations

The number of patients in our study is small and limited by ethical issues for the referral of arrhythmia-free patients to protocol-mandated repeat invasive procedure.

In line with the institutional protocol, the procedure was performed under a deep sedation, while general anesthesia was not used, which potentially could affect the ILD during WACA. Although general anesthesia (vs. deep sedation) provides better catheter stability and may decrease the PV reconnection rate at redo CA in patients with clinical recurrence of AF (26), the recent meta-analysis reported conflicting results (27).

Adenosine testing was not performed, and dormant conduction gaps could be missed in the index procedure. However, recent study demonstrated similar late PV reconnection rates between the adenosine testing and the 30-min “waiting” strategy after index PVI (28).

Esophageal temperature monitoring was not available, however alternative recommended strategies (1, 12) for preventing esophageal damage were used (see the Methods section). In addition, in previous study (9) on the LSI-guided PVI with esophageal temperature monitoring, no safety concern was reported during posterior LA wall ablation.

## Conclusion

Although the LSI-guided WACA, using the AutoMark automated tagging software, ensures a very low acute PV reconnection rate of approximately 5%, the LRs are still common. At the 3-month protocol mandated re-mapping study, the LR (of PVs) were seen in 64% of patients, 38% of WACA circles, 21% of WACA segments and 27% of PVs. However, majority of reconnected WACA segments showed only a single conduction gap, which was identified most frequently along the lateral ridge of LA. The independent risk factors for the LR of WACA circle were the left-sided location of WACA and the longer maximum ILD along WACA created in the index CA procedure. The maximum ILD of > 8 mm is a high-sensitive marker of later WACA reconnection. Our findings suggest that the availability of real time ILD feedback to operator during the index procedure could potentially improve the PVI durability after single CA.

## Data availability statement

The raw data supporting the conclusions of this article will be made available by the authors, without undue reservation.

## Ethics statement

The studies involving human participants were reviewed and approved by Ethical Committee of Clinical Centre of Serbia. The patients/participants provided their written informed consent to participate in this study.

## Author contributions

NMM and MM performed all ablation procedures, data collection, statistical analysis, and prepared manuscript. AK, VK, and VV significantly contributed in invasive ablation procedures. NaM and TP contributed to the interpretation of data and manuscript preparation. All authors contributed to the article and approved the submitted version.
